# Pacemaker programming in patients with first‐degree AV‐block: Programming pattern and possible consequences

**DOI:** 10.1002/hsr2.39

**Published:** 2018-05-09

**Authors:** F. Holmqvist, B. Rathakrishnan, L.R. Jackson, K. Campbell, J.P. Daubert

**Affiliations:** ^1^ Clinical Cardiac Electrophysiology Duke University Medical Center Durham NC USA; ^2^ Department of Cardiology Lund University Lund Sweden; ^3^ Columbia University College of Physicians and Surgeons New York City NY USA; ^4^ Department of Pharmacy Duke University Medical Center Durham NC USA

**Keywords:** first‐degree AV‐block, pacemaker programming, sinus node dysfunction

## Abstract

**Background:**

The optimal way of pacing in patients with an indication for pacing and concomitant first‐degree atrioventricular (AV)–block is not known, and consequently, firm guidelines on this topic are lacking. This study explored the current pacemaker programming pattern in patients with first‐degree AV‐block who have a dual chamber pacemaker without cardiac resynchronization.

**Methods:**

The study was a retrospective chart review conducted at Duke University Hospital. Patients receiving a pacemaker due to sinus node dysfunction with coexistent first‐degree AV‐block were studied. Baseline demographics and characteristics, as well as pacemaker programming parameters and follow‐up data, were collected through chart review. Preimplantation and postimplantation electrocardiograms were analyzed.

**Results:**

A total of 74 patients were included (mean age, 75 ± 11 y; 53% men). The mean ± SD preimplant PR interval and QRS duration was 243 ± 46 and 110 ± 30 milliseconds, respectively. A history of atrial fibrillation was present in 49% of the patients, and 77% had a normal left ventricular ejection fraction. The majority of patients (65%) had their pacemakers programmed to atrial pacing (AAI/DDD +/−R), whereas 32% and 2.7% of the pacemakers were programmed to AV‐sequential pacing (DDD) and ventricular pacing (VVI), respectively. There were no significant differences in baseline characteristics or electrocardiogram measures between patients programmed to the 3 pacing modes. Patients with pacemakers programmed to AAI had a lower ventricular pacing percentage at follow‐up (8 vs 55, and 46% [DDD and VVI, respectively]; P < .001).

**Conclusions:**

There was no evident association between baseline characteristics and programmed pacing mode in patients with first‐degree AV‐block. The choice of pacing mode affects long‐term pacing burden, which in turn has been shown to influence outcome.

## 1 INTRODUCTION

According to current guidelines, the sole presence of first‐degree atrioventricular (AV)–block is, in most instances, not an indication for pacemaker treatment.^1^ However, first‐degree AV‐block is commonly seen in patients with other indications for pacemaker therapy such as sinus node dysfunction or transient high degree of AV‐block. Optimal pacing under these circumstances is unclear, and the existing literature does not provide any firm guidance. It is well known that right ventricular pacing may have negative long‐term effects, regardless of underlying AV‐conduction.^2^, ^3^ On the other hand, atrial pacing (AAI) has been shown to be potentially detrimental when compared with either backup ventricular pacing (VVI) or AV‐sequential pacing (DDD) in patients with first‐degree AV‐block, specifically because atrial pacing at higher rates (in rate responsive modes) can further prolong the PR interval.^2^, ^4^, ^5^ This makes programming particularly challenging in patients with bradycardia when first‐degree AV‐block is part of the problem.

The present study sets out to explore how electrophysiologists at a tertiary referral center choose to program pacemakers in patients with a first‐degree AV‐block and sinus node dysfunction and whether or not there are any patient or clinical characteristics that help guide them in their decision making.

## 2 METHODS

### 2.1 Study population

We examined the medical records and preimplant electrocardiograms (ECGs) from 400 consecutive patients who had a permanent pacemaker implanted at Duke University Hospital because of sinus node dysfunction between March 2011 and December 2012 and identified patients who had a first‐degree AV‐block in addition to the sinus node dysfunction. Basic clinical characteristics and demographics were retrieved from the medical charts. The 12‐lead ECG data include computer‐generated measurements of the relevant electrocardiographic intervals (PR and RR), as analyzed using Philips TraceMaster ECG software (Andover, Massachusetts). Follow‐up data were retrieved from routine, clinical device interrogation approximately 3 months following implantation. The trial was conducted in accordance with the Helsinki Declaration and was approved by the Duke Health Institutional Review Board (Pro00049403). The requirement for informed consent was waived by the Duke Health Institutional Review Board since the research involved no risk to the subjects, the waiver did not adversely affect the rights and welfare of the subjects, and the study was conducted in a retrospective fashion.

### 2.2 Statistical analysis

Normally distributed data are expressed as mean ± SD. Median and range are used when normal distribution could not be assumed. Student *t* test was used for comparison between samples. Chi‐square was used for discrete variables. All tests were 2‐sided, and *P* < .05 was considered statistically significant. All statistical analyses were performed using IBM SPSS Statistics software (version 25 running on Mac OS X, IBM Corporation, Armonk, New York).

## 3 RESULTS

Out of the 400 screened patients, 80 (20%) were found to (1) be in sinus rhythm and (2) have a diagnosis of first‐degree AV‐block confirmed on preimplant ECG. Of those, 76 patients received a permanent pacemaker. Their mean ± SD age was 75 ± 11 years, and 53% (n = 39) were men. The mean ± SD PR interval was 243 ± 46 milliseconds, and the mean ± SD ventricular rate preimplantation was 63 ± 14 bpm. About half of the patients (n = 35, 47%) had a coronary heart disease, and 9.5% (n = 7) had congestive heart failure. Hypertension (n = 54, 73%), diabetes mellitus (n = 21, 28%), and a history of stroke or transient ischemic attack (n = 13, 18%) were common comorbidities.

Apart from first‐degree AV‐block and sinus node dysfunction, 25% (n = 18) of the patients had a history of syncope or presyncope. Thirty‐six patients (49%) had a history of atrial fibrillation, and a single patient (1.4%) had a history of supraventricular tachycardia. Seventy‐seven percent (n = 57) had a normal left ventricular ejection fraction. Most of the patients with abnormal left ventricular function (n = 12) had mildly impaired left ventricular ejection fraction (40‐54%), while one patient had moderately and another had severely impaired left ventricular function.

While a total of 48 patients (65%) had their pacemakers programmed to AAI‐pacing (or more accurately, to AAI/DDD +/−R mode switch algorithms, designed to decrease right ventricular pacing), 24 patients (32%) had their pacemakers programmed to pace in DDD‐mode, and 2 patients (10%) had their pacemakers programmed to VVI‐mode. There were no significant differences in the baseline characteristics between the different pacing programming groups apart from a less abundant history of stroke or transient ischemic attack among patients with pacemakers programmed to AAI‐pacing (Table 1). The final programmed parameters at baseline are summarized in Table 2.

**Table 1:** Differences in baseline characteristics between pacing programming groups^a^

	AAI < = > DDD	DDD	VVI	*P* value
	(n = 48)	(n = 24)	(n = 2)	
Age	75 ± 9	73 ± 13	84 ± 7	.331
Male	54%	50%	50%	.943
Coronary artery disease	46%	46%	100%	.318
Congestive heart failure	6.3%	13%	50%	.097
Normal LVEF	79%	86%	50%	.418
Valvular heart disease	13%	13%	0%	.867
Hypertension	79%	63%	50%	.246
Prior stroke/TIA	10%	25%	100%	.002
Diabetes mellitus	25%	29%	100%	.070
Paroxysmal SVT	2.1%	0%	0%	.760
Presyncope	15%	4.2%	0%	.359
Syncope	10%	17%	50%	.237
Atrial fibrillation	50%	46%	50%	.945
Ventricular rate (preimplantation)	66 ± 14	58 ± 13	56 ± 2.8	.065
PR interval (preimplantation)	242 ± 51	247 ± 35	222 ± 31	.055

Abbreviations: AAI, atrial pacing; DDD, atrioventricular–sequential pacing; AAI < = > DDD, AAI/DDD +/−R mode switch algorithms, designed to decrease right ventricular pacing; LVEF, left ventricular ejection fraction; SVT, supraventricular tachycardia; TIA, transient ischemic attack; VVI, ventricular pacing.

a Student *t* test was used for comparison between samples. Chi‐square was used for discrete variables.

**Table 2:** Programmed parameters by pacing mode

	AAI < = > DDD		DDD		VVI	
	Mean ± SD	Median (Range)	Mean ± SD	Median (Range)	Mean ± SD	Median (Range)
Lower rate (bpm)	60 ± 3	60 (50‐70)	60 ± 5	60 (60‐60)	60 ± 14	65 (50‐80)
AV interval—paced, ms	166 ± 15	180 (150‐180)	216 ± 85	240 (180‐300)		
AV interval—sensed, ms	136 ± 15	150 (120‐150)	225 ± 106	225 (150‐300)		
Upper tracking rate, bpm	128 ± 4	130 (120‐130)	130 ± 0	130 (130‐130)		
Upper sensor rate, bpm	127 ± 8	130 (120‐130)	125 ± 7	125 (120‐130)		
Mode switching rate, bpm	172 ± 5	175 (140‐175)	165 ± 7	165 (160‐170)		

Abbreviations: AAI, atrial pacing; DDD, atrioventricular–sequential pacing; AAI < = > DDD, AAI/DDD +/−R mode switch algorithms, designed to decrease right ventricular pacing; VVI, ventricular pacing.

On the postimplantation ECG (the day after the procedure), 24 patients (32%) were in sinus rhythm, whereas 28 patients (38%) were A‐paced only, 12 patients (16%) were RV‐paced (regardless of AV AV‐sequentially), and 4 patients (5%) were in atrial fibrillation. In the remaining 6 patients (8%), the ECG was missing. Heart rate preimplantation and postimplantation was higher in patients in sinus rhythm postimplantation compared with patients with atrial pacing postimplantation (72 ± 14 vs 61 ± 11 bpm, *P* = .005, and 76 ± 10 vs 62 ± 6 bpm, *P* < .001, respectively). When comparing the PR interval preimplantation between patients in sinus rhythm and with atrial pacing postimplantation, no significant difference was found (246 ± 36 vs 235 ± 39 ms, *P* = .289). The same was true for the postimplantation PR interval (237 ± 47 vs 242 ± 60 ms, *P* = .344). The delta PR was longer (ie, more pronounced prolongation) in patients with atrial pacing, but the difference was not statistically significant (−9.6 ± 36 vs 7 ± 34 ms compared with baseline, *P* = .090).

**Figure 1:**
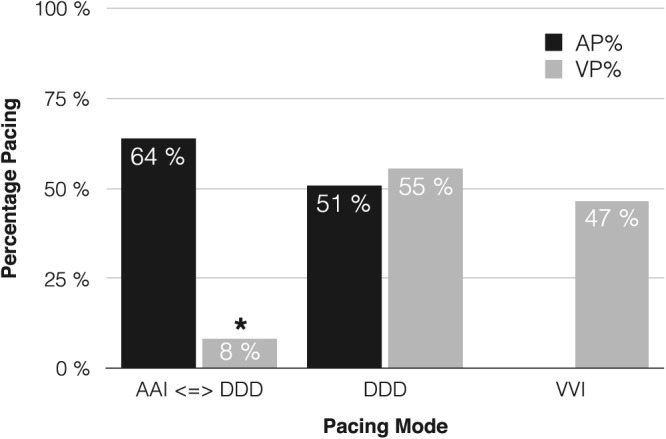
Percentage of atrial and ventricular pacing by pacemaker setting. AP, atrial pacing; VP, ventricular pacing. ^*^ *P* < .001 (chi‐square test)

At follow‐up (median, 4.9 mo; range, 1‐26 mo), the pacing percentage differed significantly depending on pacing mode, with the lowest amount of ventricular pacing seen in patients with pacemakers programmed to AAI/DDD +/−R pacing (Figure 1). The atrial fibrillation burden (ie, mode switch episodes) did not differ between patients with pacemakers programmed to AAI‐ or DDD‐pacing (5 ± 12% vs 4 ± 8%, *P* = .830).

## 4 DISCUSSION

The presence of first‐degree AV‐block in patients scheduled for a pacemaker due to sinus node dysfunction is a common finding. In the present study, one in 5 of these patients had a prolonged PR interval. The optimal way of treating patients with an indication for pacing and with a concomitant first‐degree AV‐block is presently unknown. In the DANPACE (The Danish multicenter randomised trial on single lead atrial versus dual chamber pacing in sick sinus syndrome) study, AAI‐pacing was compared with DDD‐pacing in patients with sick sinus syndrome.^6^ Patients with first‐degree AV‐block were found to fare worse than patients with normal AV‐conduction, and a higher incidence of atrial fibrillation was observed.^4^ This was particularly evident in patients randomized to AAI‐pacing. In keeping with this, analyses of the Managed Ventricular Pacing trial revealed that the presence of first‐degree AV‐block was, in essence, the driver of the negative effects observed in patients with AAI‐pacing when compared with backup VVI‐pacing.^5^ Both of these studies indicated that AAI‐pacing may be unfavorable in patients with first‐degree AV‐block. However, reports from the Dual Chamber and VVI Implantable Defibrillator trial, in which DDD‐pacing (with a high burden of RV‐pacing) was compared with VVI‐backup pacing (with a low burden of RV‐pacing) in patients with heart failure and treatment with implantable cardiac defibrillator, indicate that DDD‐pacing was worse than VVI‐backup even if the patients had first‐degree AV‐block and/or sinus bradycardia.^2^ On the other hand, it is well known that excessive pacing of the right ventricle is associated with poor outcome,^7^, ^8^ making pacing programming in patients with first‐degree AV‐block challenging.

The lack of guidance and scientific evidence is echoed in the seemingly random choice of pacing mode in patients with first‐degree AV‐block and sinus node dysfunction seen in this study. Single chamber devices (VVI‐pacing) are rarely used to treat patients with an indication for pacing and concomitant first‐degree AV‐block. One of the 2 patients with VVI devices in the current study was known to have persistent atrial fibrillation (albeit not at the time of implantation), and the other had a single chamber device implanted decades before the inclusion in the study (generator change).

Interestingly, based on the programmed parameters, one may get the impression that patients with pacemakers programmed to AAI were to a larger extent left with “out‐of‐the‐box” settings for the AV‐interval parameters (in the event of DDD‐pacing). A likely consequence of this, given the underlying first‐degree AV‐block, is a higher degree of right ventricular pacing if the AAI‐pacing ever is switched to DDD because of transient higher degree AV‐block or further PR‐prolongation, during follow‐up.

One of the mechanisms that has been suggested to explain the increase in event rate associated with AAI‐pacing in first‐degree AV‐block is an augmentation of the PR‐prolongation commonly seen with atrial pacing.^5^ A trend compatible with this phenomenon was seen in the present study, but the difference did not reach statistical significance.

Importantly, the present study shows that the choice of pacing mode in these patients is likely to have consequences. The amount of atrial and ventricular pacing differs substantially between the different pacing modes. Not surprisingly, the lowest amount of right ventricular pacing was seen in patients with a pacemaker programmed to AAI/DDD +/−R pacing. Naturally, no atrial pacing was observed in patients with pacemakers programmed to VVI‐pacing, but this comes with the price of more ventricular pacing. As mentioned above, all different pacing modes have been shown to have potential detrimental effects, and the optimal way of pacing patients with first‐degree AV‐block needs to be determined in a prospective fashion.

## 5 LIMITATIONS OF THE STUDY

The present study was based on retrospective chart review, with all of its inherent limitations. Moreover, the study data are based on the experience of a single high‐volume center, a fact that may limit the generalizability of the study.

## 6 CONCLUSION

In patients with first‐degree AV‐block and a need for pacing treated at a highly specialized tertiary cardiac electrophysiology center, there is no evident association between baseline characteristics and programmed pacing mode. The choice of pacing mode affects long‐term pacing burden, which in turn has been shown to influence outcome. The findings in the current case series highlight the need for prospective studies primarily aimed at addressing the optimal pacing mode in these patients.

## FUNDING

F.H. was funded by the Crafoord Foundation, Eva och Carl‐Eric Larsson Foundation, Bundy Academy, and Skåne University Hospital Research Foundation.

## CONFLICT OF INTERESTS

None declared.

## AUTHOR CONTRIBUTIONS

Conceptualization: Fredrik Holmqvist, Larry R. Jackson II, James P. Daubert

Funding acquisition: Kristen Campbell, James P. Daubert

Investigation: Fredrik Holmqvist, Bharath Rathakrishnan, Kristen Campbell

Methodology: Fredrik Holmqvist, Larry R. Jackson II, James P. Daubert

Resources: Kristen Campbell

Supervision: Fredrik Holmqvist, Larry R. Jackson II, James P. Daubert

Writing—original draft: Fredrik Holmqvist

Writing—review and editing: Fredrik Holmqvist, Larry R. Jackson II, Bharath Rathakrishnan, Kristen Campbell, James P. Daubert
